# System effectiveness of detection, brief intervention and refer to treatment for the people with post-traumatic emotional distress by MERS: a case report of community-based proactive intervention in South Korea

**DOI:** 10.1186/s13033-016-0083-5

**Published:** 2016-08-08

**Authors:** Mi-Kyung Yoon, Soon-Young Kim, Hye-Sun Ko, Myung-Soo Lee

**Affiliations:** 1 Gyeonggi Mental Health Center, Yongin Mental Hospital, 245 beon-gil, Jangan-gu, Suwon, Gyeonggi Province South Korea; 2 Yongin Mental Hospital, 940 Jungbudaero, Giheung-gu, Yongin, Gyeonggi Province South Korea

**Keywords:** MERS, Disaster, Post-traumatic stress, Community mental health

## Abstract

**Background:**

Korea has experienced diverse kind of disasters these days. Among them the 2015 middle eastern respiratory syndrome (MERS) outbreak imposed great psychological stress on almost all Korean citizens. Following the MERS outbreak, government is reviewing overall infectious disease management system and prioritizing the establishment of mental health service systems for infectious disease. This study makes suggestions for implementing disaster-related mental health service systems by analyzing the example of Gyeonggi Province, which proactively intervened with residents’ psychological problems caused by the large-scale outbreak of an infectious disease.

**Case description:**

Mental health service system for MERS victims had the following two parts: a mental health service for people who had been placed in quarantine and a service provided to families of patients who had died or recovered patients. The government of Gyeonggi province, public health centers, regional and local Community Mental Health Centers and the National Center for Crisis Mental Health Management participated in this service system. Among 1221 Gyeonggi people placed in quarantine and who experienced psychological and emotional difficulties, 350 required continuing services; 124 of this group received continuing services. That is, 35 % of people who required psychological intervention received contact from service providers and received the required services.

**Conclusions:**

This study reflects a proactive monitoring system for thousands of people placed under quarantine for the first time in Korea. It is significant that the service utilization rate by a proactive manner, that is the professionals administering it actively approached and contacted people with problems rather than passively providing information was much higher than other general mental health situation in Korea. The core value of public mental health services is adequate public accessibility; it is therefore essential for governments to strengthen their professional competence and establish effective systems. These criteria should also be applied to psychological problems caused by disastrous infectious disease outbreaks.

## Background

Gyeonggi Province in South Korea is a regional governmental body with a population of 12 million people; it is composed of 31 municipalities. Natural and social disasters of various severity have occurred in Gyeonggi Province; in 2014, 26.5 % of all natural disasters in Korea occurred in Gyeonggi Province [1, 2]. Among such disasters, the 2015 middle eastern respiratory syndrome (MERS) outbreak imposed great psychological stress on almost all Korean citizens. One hundred and eighty-six South Koreans were infected by MERS; 38 died and 146 recovered. A further, 16,752 were placed in quarantine [3]. In Gyeonggi Province, where the first MERS patient was discovered in Korea, 70 people were infected by MERS (37 % of Korean MERS patients), eight died (21 % of MERS deaths), and 11,318 were monitored or placed in quarantine (67 % of the quarantined population).

People who recover from infectious diseases with a high mortality rate such as MERS, often show emotional instability resulting from the experience of facing death [4]. Family members of people killed by such diseases also often become depressed following the sudden loss [5]. Risk communication regarding infectious diseases with a high mortality rate tends to arouse public anxiety and fear [6, 7]. The National Disaster Management Institute examined South Koreans’ emotional responses on social networking services during the MERS outbreak; those responses were as follows: despair during the first 9 days of the outbreak, anxiety on the 15th–19th days, and anger on the 20th–31st days [8]. Over a quarter of Hong Kong residents who experienced the severe acute respiratory syndrome (SARS) outbreak showed symptoms of chronic fatigue syndrome (27.1 %); 42.5 % reported experiencing psychological problems 4 years after the outbreak [9]. Patients infected by the Ebola virus also experienced post-traumatic stress disorder, depression, anxiety disorder and survivor guilt [10]. Families and friends whose loved ones were killed by the Ebola virus disease experienced psychological problems, including depression, sleep disturbances, abnormal behavior and post-traumatic stress symptoms, additionally, their use of alcohol and nicotine increased following the loss [11].

The healthcare system provided mental health services to government employees and livestock farmers who were required to kill animals due to foot-and-mouth disease [12–14]; however, South Korea has never operated mental health services for infectious diseases affecting humans. The Korean health management system reacted relatively well to the Ebola and SARS viruses (which also caused major infectious outbreaks); little motivation has therefore existed to implement psychological interventions. Gyeonggi Province’s proactive intervention with MERS victims constitutes part of the national reinforcement of disaster mental health services, which have recently attracted increased attention. Particularly, following the 2014 Sewol Ferry accident (which resulted in the fourth highest number of deaths), the national and local governments have worked to establish disaster mental health service systems, and Gyeonggi Province, which has experienced various disasters, is participating particularly actively.

In order to provide an effective disaster mental health service, it is important to find professional experts and establish good programs; moreover, it is also essential to establish sensible governance between central and local governments, between administrative institutions and institutions that provide services, and between public and civil organizations. In Korea, the law has been amended following the Sewol Ferry accident, and the Ministry of Public Safety and Security now controls disaster response. Seventeen Psychological Support Centers for Disasters affiliated with the Ministry of Public Safety and Security are being operated in South Korea. Moreover, the National Center for Crisis Mental Health Management (NCMHM) is located in a national mental hospital affiliated with the Ministry of Health and Welfare, and 15 regional mental health centers and 209 local Community Mental Health Centers (CMHCs) are providing services addressing various mental health problems. The Ministry of Public Safety and Security’s system and that of the Ministry of Health and Welfare occasionally over-lap or operate in cooperation. Additionally, regarding mental health support for MERS, the two ministries took different roles based on the current situation and provided required services in each affected region. In Gyeonggi Province, Psychological Support Centers for Disasters and regional mental health centers are operating in concert.

This study makes suggestions for implementing disaster-related mental health service systems tailored to disasters’ type and timing, by analyzing the example of Gyeonggi Province, which proactively intervened with residents’ psychological problems caused by the large-scale outbreak of an infectious disease.

## Case description

On June 17, 2015, 28 days after the discovery of the first MERS patient, the government of Gyeonggi Province planned a mental health service program for people that had been directly affected by MERS. Regional and local CMHCs mostly provided Gyeonggi Province’s mental health service for MERS victims under administrative support from the Gyeonggi Province government and Public Health Centers (PHCs). In particular, regarding psychological evaluation and intervention with people who had been placed in quarantine, the Gyeonggi Province government prepared a special budget and dispatched additional employees to nine municipalities; moreover, the government developed and distributed video materials and online-based psychological stabilization programs in order to aid the psychological stabilization of other people not directly affected by MERS.

**Fig. 1:**
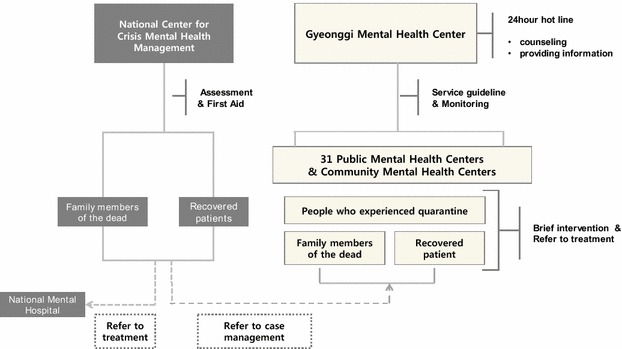
Psychological intervention system for MERS victims

This mental health service system for MERS victims had the following two parts: a mental health service for people who had been placed in quarantine; (this was conducted cooperatively between Gyeonggi Province, 31 municipal public health centers, and community mental health centers, and a service provided to families of patients who had died or recovered patients. The National Center for Crisis Mental Health Management evaluated people using these services and subsequently transferred them local CMHCs for continuous case management and follow-up (Fig. 1).

By the law of prevention and management of infectious disease, government should monitor the patients and the people in quarantine. The study sample and primary data about mental health status were collected by the government of Gyeonggi Province. We received the secondary data from Gyeonggi government and analysed those results.

### Systems for people who experienced quarantine

Each PHC monitored quarantined individuals’ physical symptoms twice daily. PHCs provided information to quarantined individuals during monitoring concerning managing psychological difficulties that might arise during quarantine and using the consultation service. CMHCs also psychologically evaluated all quarantined individuals during monitoring. The mental health professionals of CHMCs asked key questions about depression, “for the last 2 weeks or after being in quarantine, do you feel depressed or hopelessness? Do you feel loss of interest in any part of your life?” After the key questions, they provided psychological support, psycho-educational approach and providing information.

Six thousand two hundred and thirty-one people were evaluated; 6157 individuals was evaluated via phone call (99.6 %), outreach evaluation (33 cases; 0.5 %), and on-site evaluation (18 cases; 0.3 %). Initially, CMHC staff were only able to evaluate and consult quarantined individuals via phone call due to quarantine requirements. Moreover, many individuals were unwilling to provide personal information due to anticipated stigma or negative local perception of MERS, further raising the proportion of telephone consultation.

**Fig. 2:**
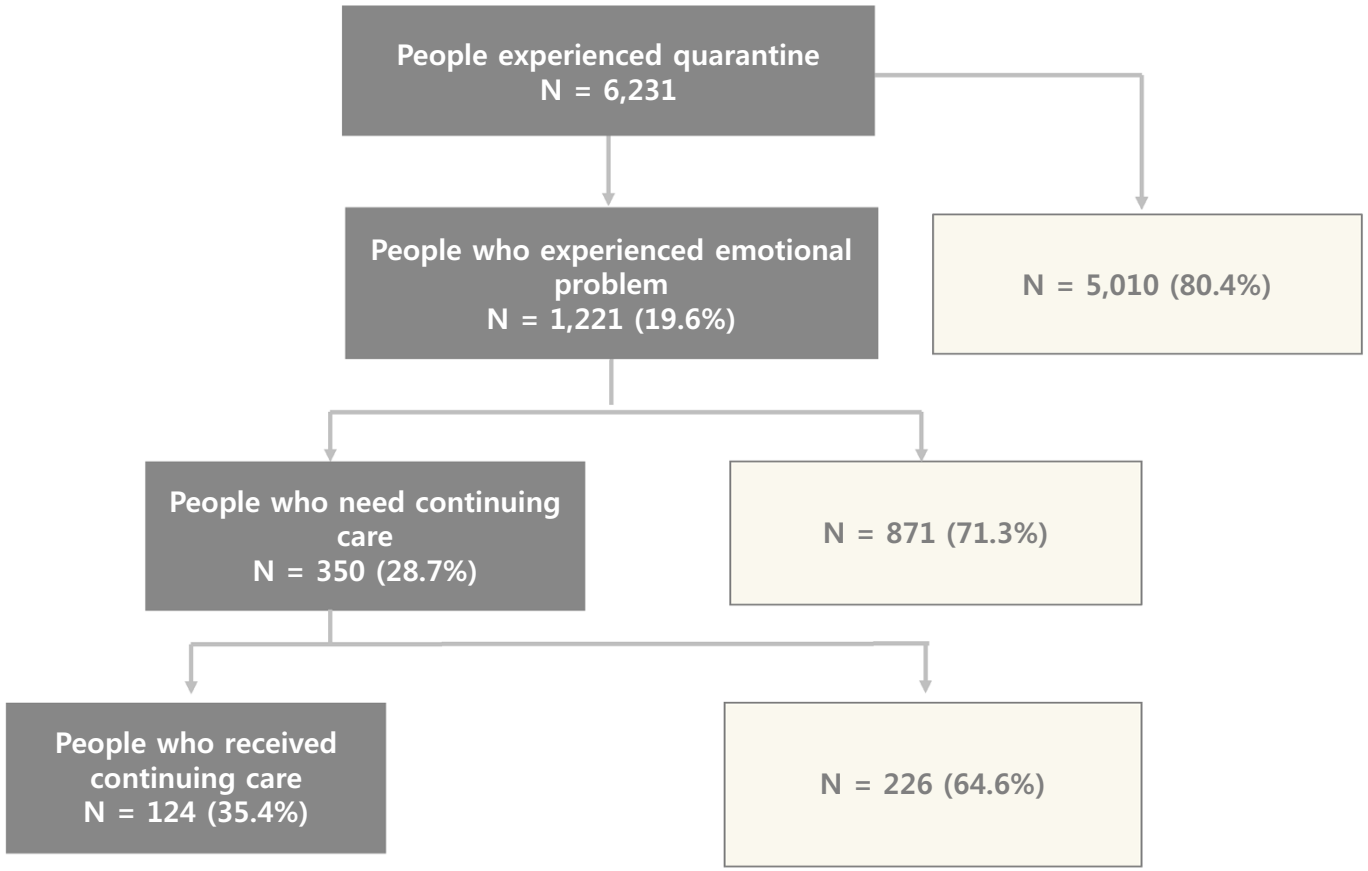
Service algorithm and numbers of clients on each level of process

Of the 6231 people placed in quarantine, 1221 showed emotional disturbances such as depression (19.3 %). Of this population, 871 (71.3 %) received only one consultation and 350 (28.7 %) required continuing services. Among the latter group, 124 (35.4; 2.0 % of quarantined individuals) received continuing services; the remaining 226 could not be reached (64.6; 3.6 % of quarantined individuals). The 124 people who received continuing services received an average of 4.7 consultations; 51 individuals in this group received more than one face-to-face service (41, 0.8 % of quarantined individuals) and CMHC staff evaluated five of these 51 individuals as facing a high-risk of mental illness and referred them to psychiatric treatment (Fig. 2).

### Systems for recovered MERS patients and family members of deceased patients

The NCMHM conducted the initial evaluation of all deceased patients’ family members and recovered patients. The NCMHM referred individuals who required further medical treatment to five national mental hospitals and those who required continuous mental health services to local CMHCs; however, of those who used the disaster mental health service in Gyeonggi Province, the NCMHM referred only four of 37 (10.8 %) deceased patients’ family members and 11 of 62 (17.7 %) recovered patients. This means that in terms of disaster mental health service system, a considerably higher number of family members and recovered patients used the Gyeonggi Province service directly, rather than the national system. This result shows us that the referral system from the national level to regional or local level does not working and it would be more efficient to develop open entry system at the local level rather than linear triage system.

## Conclusions

This study was done in a real world not an experimental situation. Mental health service interventions for MERS victims were performed by the law not by the research design. We tried to find out the way how the system works for MERS victims, systematic characteristics how the victims use mental health services, the needs of victims and the possible strategy how to develop mental health service system in the near future especially for the disaster by infectious disease.

Nonetheless, it remains significant that the service utilization rate analyzed in this study was much higher than other general mental health situation in Korea; the professionals administering it actively approached and contacted people with problems rather than passively providing information. The need for mental health services is increasing and traditional systems centered on hospitals and medical facilities are ill-suited to addressing many mental health problems. The core value of public mental health services is adequate public accessibility; it is therefore essential for governments to strengthen their professional competence and establish effective systems. These criteria should also be applied to psychological problems caused by disastrous infectious disease outbreaks.

## Abbreviations

MERS: middle eastern respiratory syndrome
NCMHM: The National Center for Crisis Mental Health Management
CMHCs: Community Mental Health Centers
PHCs: Public Health Centers
